# The Impact of Job Stress and State Anger on Turnover Intention Among Nurses During COVID-19: The Mediating Role of Emotional Exhaustion

**DOI:** 10.3389/fpsyg.2021.810378

**Published:** 2022-02-09

**Authors:** Syed Haider Ali Shah, Aftab Haider, Jiang Jindong, Ayesha Mumtaz, Nosheen Rafiq

**Affiliations:** ^1^Department of Business Studies, Bahria University, Islamabad, Pakistan; ^2^Department of Psychology, Jinhenyi School of Education, Hangzhou Normal University, Hangzhou, China; ^3^College of Public Administration, Zhejiang University, Hangzhou, China; ^4^Department of Management Sciences, Bahria University, Islamabad, Pakistan

**Keywords:** COVID-19, job stress, state anger, emotional exhaustion, turnover intentions, social exchange theory, nurses, Pakistan

## Abstract

Based on the social exchange theory, the aim of this study is to identify the association between job stress state anger, emotional exhaustion and job turnover intention. This study postulates that job related stress and state anger among nurses during COVID-19 subsequently leads to their job turnover intentions. In addition, the study also aims to see the mediating role of emotional exhaustion between COVID-19-related job stress, state anger, and turnover intentions. The sample of this study is gathered from 335 registered nurses working in Pakistani hospitals dealing with COVID-19-related patients. The interrelationships between variables are checked by using structural equation modeling through AMOS. Key findings confirm that COVID-19-related job stress and state anger had a significant effect on nurses’ turnover intentions. Furthermore, emotional exhaustion mediated the relationship between COVID-19-related job stress, state anger, and turnover intentions. There is a lack of research which has assessed the impact of Novel COVID-19-related job stress and state anger on nurses’ turnover intentions in hospitals, providing empirical evidence from a developing country-Pakistan. This study offers managerial implications for hospital management and health policymakers. Moreover, nursing managers need to pay attention to nurses’ turnover intentions who are facing the issue at the front line as patients receive their initial treatment from nurses in the COVID-19 outbreak.

## Introduction

The Novel Coronavirus 2019, currently referred to as COVID-19, is making rounds and has been in the limelight since the beginning of 2020 (Chinese Center for Disease Control and Prevention Epidemiology Working Group for NCIP Epidemic Response, 2020; World Health Organization, 2020). Although the coronavirus has existed for many years, COVID-19 is a new strain, which caused great panic around the world, affecting the general public (Human Coronavirus Types, 2020), causing damage to public health, and loss in financial and economic sectors globally (Lin et al., 2021). People with compromised immunity are prone to this new strain of coronavirus. The COVID-19 pandemic causing a severe acute respiratory syndrome (SARS) Coronavirus 2 (SARS-CoV-2) infection (Rehman et al., 2021). More than 81,000 patients have been identified thus far as being positive for COVID-19 (Mossa-Basha et al., 2020). Thus, the World Health Organization declared it a global health emergency on January 30, 2020 (Mahase, 2020). The nature of the emergency caused hundreds and thousands of health workers to be deployed to the infected areas to rescue, manage, and control the infection of COVID-19.

Turnover intention is defined as an employee’s own estimated probability of leaving his or her job or current organization at some point in the near future permanently due to various factors (Khan et al., 2020). Nurses working in the emergency departments are the first line of defense since most infected patients approach the emergency department (Rafiq et al., 2020). Their job nature requires 24/7 care delivery, and is an appropriate representative of emergency frontline professions. Due to contagious nature of COVID-19, nurses are the most exposed to this novel infection (Osterdahl et al., 2020), spend much effort in caring for infected patients and frequently experience related psychological health problems (Mumtaz et al., 2021; Xu X. et al., 2021). Such health problem can negatively impact quality of patient care (Maharaj et al., 2019) and increase their job turnover intention (Sasso et al., 2019), and puts them under persistent job stress (Cepale et al., 2021). Consequently, many nurses intend to quit their organizations (Potter, 2006; Adriaenssens et al., 2015; Shah et al., 2021). Studies on nursing have shown a strong association between job stress and nurses’ turnover intentions, arguing that job stress actually triggers nurses’ turnover intentions (Gates et al., 2011; Anees et al., 2021; Shah et al., 2021). This notion is supported by other research studies where job stress has been found to activate emotional exhaustion, which includes a negative self-concept and work attitude, resulting in a loss of interest in patients and increased turnover intentions (Sonnentag et al., 2010; Lapointe et al., 2011). Employees’ turnover is a matter of concern for organizations, and results in a loss of knowledge gained by the employee during the job period (Bajrami et al., 2021). Understaffing is another concern which in turn leads to decreased productivity and effectiveness of the remaining staff (Jha, 2009). It has also additional costs related to recruitment and selection, training of new employees, personnel process, and induction, especially during emergency situations.

State anger as a result of the perceived COVID-19 threat remained unexplored in empirical researches, and mostly discussed in qualitative studies on nurses’ experiences previously (Maunder et al., 2003; Robertson et al., 2004; Mok et al., 2005; Fiksenbaum et al., 2007). According to Spielberger (1999a, p. 1), state anger is defined as “a psychobiological emotional state or condition marked by subjective feelings that vary in intensity from mild irritation or annoyance to intense fury and rage.” State anger in organizational behavior literature is referred to as an indicator of increased workload, erosion of the psychological contract, and job deterioration (Greenglass et al., 2001, 2003; Shah et al., 2016). Research studies have shown a strong positive association between state anger and anxiety, psychoticism, and emotional exhaustion (Spielberger, 1999b; Fiksenbaum et al., 2007; Li et al., 2017; Shah et al., 2021). The COVID-19 pandemic has raised the level of job stress and uncertainty among nurses for the same reason that levels of state anger lead to greater levels of emotional exhaustion, increasing the turnover intentions of nurses (Robertson et al., 2004; Fiksenbaum et al., 2007; Özdemir and Kerse, 2020).

Nurses who are working with COVID-19 patients are more vulnerable to infection and carry fatal risks. Previous research studies on SARS show that nurses who were quarantined reported greater levels of: frustration, anger, loss of control, and perceived stigmatization (Robertson et al., 2004; Brossoit et al., 2020; Rahaman et al., 2020); post-traumatic stress disorder (Hawryluck et al., 2004); and anxiety, interpersonal rejection, fear of infecting others, and social isolation (Maunder et al., 2003; Bai et al., 2004; Nickell et al., 2004; Fiksenbaum et al., 2007). The global breakout of COVID-19 is a traumatic event, unfamiliar even to nurses because there is little research on COVID-19-related job stress and state anger. Moreover, with the widespread infection of COVID-19, many nurses are experiencing job stress (De los Santos and Labrague, 2021) and state anger, with some nurses feeling reluctant to care for patients (Brug et al., 2004; Shiao et al., 2007). Similarly, such factors like job stress and state anger trigger nurses’ emotional exhaustion, which leads to an increased desire to leave their jobs (Shiao et al., 2007; Zhang et al., 2020). A previous study from Taiwan during the breakout of the H5N1 avian flu, found that nurses working in Taiwanese hospitals were experiencing fear of infection and job stress, which were found to be significant factors in dealing with infected patients of avian flu (Tzeng and Yin, 2006; Teng et al., 2021). Due to the emergence and spread of COVID-19 worldwide; nurses are the key actors from the emergency team to understand the criticality and breadth of patient care needs to address most efficiently (Arasli et al., 2020). Therefore, nurse’s turnover has a significant impact on the organization and leaders who desire to preserve a seasoned and competent nursing workforce (Harun et al., 2020; Nemteanu and Dabija, 2021). Nursing Turnover is a serious concern for healthcare provision during pandemics. It has an impact on quality of patient care provision, continuity of care interruption, loss of skill full and knowledgeable staff and additional time and financial costs for replacement and training of staff. It also has a negative impact for remaining staff, related with morale and increased workload leading to achieve goal of providing instant quality care to infected people (Price and Mueller, 1981; Kiani et al., 2020).

This study also extends the literature by examining the relationship of emotional exhaustion between job stress and job anger on turnover intentions during the pandemic of COVID-19. Results of the study may contribute to understand the phenomena of such job anger and job stress during the covid-29 (Cropanzano et al., 2003; Karatepe, 2013). This study paved the way for better understanding of the mechanisms when unfavorable conditions contribute to an employee’s turnover intentions (Hur et al., 2015). Moreover, Job stress along with emotional exhaustion contributes to a wide array of withdrawal responses (Howard and Cordes, 2010) which severely effect the health industry. The significance of these findings in extending social exchange theory concepts to the field of employee turnover intentions is examined (Biron and Boon, 2013). Finally, we contribute to the pandemic covid19 related literature by providing an empirically evidences derived from a unique framework and by demonstrating that less job stress and job anger caused by COVID-19 contribute to emotional exhaustion in much the same way that trigger the employee turnover intentions and emotional suffering.

Understanding more about the interrelationships between different factors affecting turnover intention can be used by the emergency departments and hospital managements to develop policies and practices designed to increase job retention among nurses. Nursing managers need to pay attention to nurses’ job stress, state anger, and emotional exhaustion in association with their experiences of the nationwide COVID-19 breakout (Soto-Rubio et al., 2020).

Even if there are limited studies on nurses’ turnover Intention, they focused on turnover intention in normal situations (Lee and Kim, 2020; Yao et al., 2020). There are no studies conducted on nurse’s turnover intentions due to job stress and state anger experienced during COVID-19. There is need of research in this area which was initiated to this study. Moreover, this study adding the literature by assessing the role of nurses’ emotional exhaustion triggered by job stress and state anger. Thus, this study contributes to the body of knowledge by integrating disperse scholastic work into one framework (Xiang et al., 2020). The proposed research contributes theoretical and empirically to the organizational management research. This study provides the insights on the job turnover intentions due to stressors during emergency situations; such as COVID-19 in Hospital nurses, and provides further implications for the nursing managers and hospital management to deal with the staff during pandemics.

The following research questions are proposed for the current research study based on above mentioned gaps,

*RQ1. Does COVID-19 related job stress have an effect on employee turnover intentions during the pandemic?*

*RQ1. Does COVID-19 related state anger have an effect on employee turnover intentions during the pandemic?*

*RQ2. Does emotional exhaustion mediate the relationships among COVID-19 related job stress, state anger and employee turnover intentions?*

## Literature Review and Hypothesis Development

### COVID-19-Related Job Stress and Turnover Intentions

Clegg (2001) defines job stress as a condition of strain that creates tension in an individual. During the COVID-19 pandemic, increasing numbers of suspected cases are reaching hospitals, while the capacities of healthcare institutions are limited in treating patients, which is causing job stress among healthcare professionals (Sultana et al., 2020). Moreover, during pandemic, healthcare professionals are also concerned about their families, which impact their psychological health (Rana et al., 2020). Job stress is considered as one of a consequence of COVID-19 (Khalatbari-Soltani et al., 2020) which has an impact on the nursing profession. The nursing profession is considered highly stressful and challenging because of the high complexity involved, requiring expertise and needing to handle emergency situations with great care (Chen et al., 2009). Stress occurs in those individuals and groups of individuals who interact with highly complex situations, which are ambiguous, demanding, and unclear with regard to competence (Bass, 1985). Further, literature shows that nurses are the most affected group within healthcare organizations who become the victims of job stress (Hong et al., 2021; Shah et al., 2021). Furthermore, as Trybou et al. (2014) suggest, job stress can easily be seen among nurses. In addition, a study conducted by Chen et al. (2018) on intensive care units (ICUs) found that nurses suffer more severe job stress due to patients’ severe health conditions in the ICU.

Literature shows that there is another important variable that is highly influenced by job stress – turnover intentions (Lee et al., 2013; Shah et al., 2021). Jones (1990) suggests that turnover is the withdrawal from a job by an individual from their workplace. Turnover is defined as the voluntary withdrawal of an individual from his or her workplace (Morrell, 2005).

Limited studies have been conducted to assess the relationship between job related stress and turnover intention. Chen et al. (2009), Zhang et al. (2020), and Said and El-Shafei (2021) found that job stress has a direct correlation with turnover intentions among nurses. It has also been observed that, during different kinds of viral spreads, nurses become the victims of job stress and they prefer to leave their jobs (Haas et al., 2020). A research study, for example, conducted in South Korea, in which post-traumatic stress and nurses’ turnover intentions were measured during an outbreak of Middle East Respiratory Syndrome (MERS), suggests that post-traumatic stress disorder is positively associated with nurses’ turnover intentions (Jung et al., 2020). Moreover, Choi and Kim (2020) conducted a study on Korean nurses’ job stress and their turnover intentions during the control of infection. They found that job stress is a significant predictor of turnover intentions. Chen et al. (2005) conducted a study on Taiwanese nurses who were taking care of patients suffering from SARS in 2003. These nurses were suffering from severe post-traumatic stress compared to those who were not directly involved in taking care of SARS patients. On this basis, the following hypothesis is proposed:

H1: COVID-19-related job stress has a direct positive association with turnover intentions.

### COVID-19-Related Job Stress and Emotional Exhaustion

Job stress is the phenomenon that is common among healthcare experts – especially among nurses during the COVID-19 pandemic. The increased number of cases caused mental stress among nurses (Mo et al., 2020). Mental stress causes a negative impact on nurses’ physical and mental health (Oktug, 2017). Due to COVID-19, a wide range of job stressors are being observed among healthcare professionals (Sultana et al., 2020). Job burden and increasing job demands are causing job stress, leading to emotional exhaustion among nurses (Barello et al., 2021). Emotional exhaustion is defined as the feeling of being drained as a result of heavy workloads in the workplace (Zhang et al., 2021). The COVID-19 has posed the most serious respiratory virus threat to the employees working in a health sector, since the 1918 influenza pandemic (Ferguson et al., 2020). During the COVID-19 pandemic, as overworked hospitals are faced with the flow of confirmed and suspected cases, the shortage of health care workers (Yang et al., 2021). The influence of COVID-19 on psychological health and work-related outcome has been remained a concern and an issue of interest for researchers recently (Islam et al., 2021; Khan, 2021a; Nadeem and Khaliq, 2021). Since the beginning of the pandemic the medical personnel have played a critical role in crises and particularly in situation of emergencies around the world (Khan, 2021b).

Job stress creates mental disturbances among nurses due to the higher rate of pandemic spread, shortage of healthcare facilities, the careless attitude of the general public, an imbalance between family-life and work-life, and uncertainty about pandemic-related future situations (Serrão et al., 2021). All these situations cause emotional exhaustion among nurses. Indeed, the literature has proven that job stress has a significant positive influence on emotional exhaustion among nurses (Maslach and Jackson, 1981). Moreover, employees with heavy work load experience tiredness and exhaustion (Mulki et al., 2006). Further, Maslach and Jackson (1981) proved that emotional exhaustion is one of the most significant components of burnout, while the other two components are personal accomplishments and depersonalization. Another study conducted in China showed that Chinese nurses are more prone to emotional exhaustion due to their involvement in stressful tasks (Ding et al., 2015). In addition, Oktug (2017) advocates that job stress brings emotional exhaustion among nurses. Following the same notion, another study showed a strong association between job stress and emotional exhaustion (Arnsten et al., 2015). On the basis of the above discussion, the following hypothesis is proposed:

H3: COVID-19-related job stress has a direct positive association with emotional exhaustion.

### COVID-19-Related Job Stress, Turnover Intentions, and Mediation of Emotional Exhaustion

When job stress causes emotional exhaustion and emotional suffering, thus outbreak the turnover intentions, emotional exhaustion is indirectly contributing (Karatepe, 2013). Experiencing these job stress and state anger, thus depleting emotional and cognitive resources and contributing to emotional exhaustion. Some studies have found evidence for a link between job stress and job anger and emotional exhaustion. For example, Emotional exhaustion is a form of strain associated with these stressors (Cropanzano et al., 2003). Emotional exhaustion has emerged as the core or central dimension of burnout and job stress (Jackson et al., 1986) based on research identifying it as the job stress component that is most responsive to stressors in the work environment (Gaines and Jermier, 1983; Leiter, 1991; Saxton et al., 1991; Lee and Ashforth, 1993; Wright and Cropanzano, 1998; Cropanzano et al., 2003; Karatepe, 2013). A study that investigated the framework of the job demands resources model and employee anger in china and collected data from 411 offices working in five different cities, and they found the associations between jib stress and emotional exhaustion (VanYperen et al., 1992; Li et al., 2017). Gaines and Jermier (1983) also investigated the perceptions of pay inequity in various departments of # police bureau were found to be related to emotional exhaustion. Moreover, injustices, workload and job stress, such as jobs that fail to result in desired benefits (Cropanzano et al., 2003), have been associated with emotional exhaustion (Kahn and Byosiere, 1992; Moore, 2000). The study was conducted with a sample of 445 nurses and health care assistants from a general hospital. Four of their specific job stressors were taken into account (workload, patients’ and relatives’ requirements, patients’ suffering, and team collaboration problems), results indicated that coworkers stress were related to emotional exhaustion and burnout (Andela et al., 2016; Koon and Pun, 2018). Since the link of job stress and job anger has been found in the literature and there is gap and need to investigate the mediating role of emotional exhaustion. Owing to this gap, this study intends to investigate the mediating role of emotional exhaustion.

Few studies have been conducted to investigate the association between job stress, emotional exhaustion, and turnover intentions (Price and Mueller, 1981; Jha, 2009; Labrague et al., 2021). An increased level of stress impacts a worker’s performance and negatively influences their behaviors (Gilboa et al., 2008). A study by Noor and Maad (2008) shows the association between job stress and turnover intentions. Furthermore, Mxenge et al. (2014) also showed that job stress strongly influences turnover intentions among employees. Many research studies have argued that if an organization wants to retain its employees, it should reduce job stressors at the workplace (Kaur et al., 2013; Khan et al., 2014).

Emotional Exhaustion happens when an individual is facing overstretched demands and time (Labrague and de Los Santos, 2021). Similarly, people who are emotionally exhausted will desire to eliminate themselves from depleting workplace, creating a higher turnover intention and an more intentions toward withdrawal from organization. Past empirical studies have indicated the significant support of emotional exhaustion as a predictor to many critical outcome variables, for instance turnover intentions (Catton, 2020; Mo et al., 2020). An extensive review of literature concluded that emotional exhaustion happens due to lack of support and autonomy, overstretch conditions, role ambiguity and so on (Gkorezis et al., 2015; Blau, 1964). Front-line health workers are more susceptible to emotional exhaustion. COVID-19 pandemic has increased the probability of emotional exhaustion in health care workers due to high stress and overload (Kang et al., 2021). An empirical study has examined that front-line medical workers who are directly dealing with COVID-19 patients are more likely to experience emotional exhaustion. As a result, emotional exhaustion in front-line health workers is more associated with a variety of occupational stress (Wu et al., 2021).

Moreover, many studies have also shown that employees who suffer emotional exhaustion are more inclined to leave their workplace (Maslach et al., 2001). Similarly, Huang et al. (2010) showed that emotional exhaustion has a positive association with employees’ turnover intentions. Likewise, Choi et al. (2012) showed in their study that emotional exhaustion acts as a moderator among job stress and turnover intentions among nurses. Our study suggests that COVID-19-related conditions cause job stress among nurses, which triggers emotional exhaustion, consequently increasing turnover intentions.

According to Maslach and Jackson (1981), job stress causes burnout among employees – especially emotional exhaustion. In addition, for healthcare professionals, emotional exhaustion is an occupational disease that impacts their physical and mental health (Tziner et al., 2015), leading to increased turnover intentions (Babakus et al., 2008; Reb et al., 2017). Likewise, Azharudeen and Arulrajah (2018) conducted a study and showed that job stress and emotional exhaustion influence turnover intentions. However, there are also some studies that have found a non-significant association of job stress and turnover intentions. For instance, a study by Tziner et al. (2015) found no association of job stress on turnover intentions, while another study by Bedeian and Armenakis (1981) showed the same results, that job stress and turnover intentions have weak associations with each other. Moreover, Günüşen et al. (2014) conducted a study in which they showed that work stress positively influences emotional exhaustion among nurses. Based on the above discussion, the following hypothesis is proposed:

H6: Emotional exhaustion mediates the relationship between COVID-19-related job stress and turnover intentions.

### State Anger and Turnover Intentions

State anger is the result of the current COVID-19 pandemic and its prevailing impacts. Due to spread of COVID-19, nurses’ concerns regarding their work and its impact on their family members build psychological suffering among nurses, which causes state anger (Bayrak et al., 2021). State anger refers to a psychobiological state in which an individual’s emotions fluctuate from minor irritation to an intense level of rage (Spielberger, 1999a). In the literature, the association of state anger has been investigated with many other variables, such as depression, anxiety, and job stress (Grandey et al., 2002; Kiecolt-Glaser et al., 2002; Sloan, 2004; Khan et al., 2020; Shah et al., 2020). The literature suggests that interpersonal interactions with others positively influence state anger among employees, which leads them toward intentions to leave (Sloan, 2004). According to Kiecolt-Glaser et al. (2002) and Nemati et al. (2020), anger causes psychological disturbances among individuals, which weakens their immune system and causes death. Thus, state anger increases individuals’ intentions to leave the job. Moreover, Booth and Mann (2005) also found a strong association between state anger and turnover intentions. However, there are also some studies that do not suggest strong associations between state anger and turnover intentions (Greenglass, 1987; Grandey et al., 2002; Harlos, 2010). According to Grandey et al. (2002), there is no strong association between state anger and turnover intentions. Harlos (2010), however, supported the association between state anger and turnover intentions. In addition, Greenglass (1987) found state anger has a positive influence on turnover intentions. On the basis of the above discussion, the following hypothesis is proposed:

H2: COVID-19-related state anger has a direct positive association with turnover intentions.

### State Anger and Emotional Exhaustion

It is almost definite that nurses will suffer emotional exhaustion caused by state anger in the current situation; COVID-19 creates uncertainty in nurses, so it is obvious to expect anger due to this outbreak. However, limited studies have been conducted on state anger and emotional exhaustion among nurses. Due to this pandemic, state anger is likely to cause uncomfortable and unpleasant reactions among employees (Goldman, 2003). Moreover, anger motivates staff to attack others due to emotional exhaustion. Emotional exhaustion is one dimension of burnout. Burnout is a situation in which a loss of desire to work occurs (Booth, 2021). Once aroused, these negative emotions make nurses emotionally exhausted. When nurses suppress their anger, it affects their physical and psychological health. Unpleasant emotions of frustration, anger, and unhappiness contribute toward burnout (Carson, 2006). Moreover, Greenglass et al. (2003) and Puiu et al. (2020) advocate that state anger is an indicator of distress, which is caused due to workload. They showed that state anger mediates the association between workload and depression among nurses. Similarly, another study showed that anger has a positive relationship with depression, neuroticism, and anxiety (Spielberger, 1999a). Further, the literature shows that an association between burnout predicts personal distress, anxiety, and depression (Greenglass et al., 2001). Chang (2009) also showed in their study that unpleasant emotions are significantly related to burnout. Unpleasant emotions, such as state anger, cause emotional exhaustion among employees (Bullough et al., 2006; Puiu et al., 2020; Chen and Eyoun, 2021). When nurses suffer high job demands, it causes anger and aggression among employees. This anger exaggerates in the form of emotional exhaustion. This study assumes an association between state anger and emotional exhaustion on the basis of the above discussion, and proposes the following hypothesis:

H4: There is a positive relationship between COVID-19-related state anger and emotional exhaustion.

H5: There is a positive relationship between emotional exhaustion and turnover intentions.

### COVID-19-Related State Anger and Turnover Intentions With Emotional Exhaustion as a Mediator

The impact of state anger and emotional exhaustion on turnover intentions is, receiving increased attention during the COVID-19. Literature shows that state anger impacts employees’ turnover intentions. According to Wright and Cropanzano (1998), state anger increases turnover and absenteeism among employees. Harlos and Axelrod (2008) conducted a study in which he showed that employees who face verbal abuse are unable to do their work effectively, and they plan to leave their jobs. Griffeth et al. (2000) found that negative psychological events lead individuals toward voluntary turnover. There are also some studies, however, which show contrary results. According to Grandey et al. (2002), for example, there is no association between anger and turnover intentions. According to Kiecolt-Glaser et al. (2002), state anger creates psychological distress that causes disease and death and is positively related to turnover intentions.

Previous studies show the association between state anger and emotional exhaustion (Fiksenbaum et al., 2007; Lim et al., 2020). This anger suppression creates emotional exhaustion among employees. Furthermore, Kraemer and Gouthier (2012) showed that state anger is the reason for emotional exhaustion among employees. This emotional exhaustion increases turnover intentions among employees. In addition, literature has also proven the association between state anger and turnover intentions (Karatepe and Karatepe, 2009; Jiang et al., 2017). In the same way, Ducharme et al. (2007) conducted a study and showed that emotional exhaustion is a significant predictor of intention to quit the job. Lloyd et al. (2015) showed that emotional exhaustion predicts the correlation with turnover intentions. Based on the above discussion, the following hypothesis is proposed:

H7: Emotional exhaustion mediates the relationship between COVID-19-related state anger and turnover intentions.

### Theoretical Framework

Social exchange theory was developed by George Homans, a sociologist. Social Exchange Theory proposes that behaviors can be thought of as the result of cost-benefit analyses by people attempting to interact with society and the environment (Homans, 1958). It first appeared in his essay “Social Behavior as Exchange,” in 1958. Social behavior is theorized as an exchange of material and non-material goods, like time, money, effort, approval, prestige, power, etc., this theory assumes that social behavior is determined by the interaction of two variables after a cost–benefit analysis.

The significance of these findings in extending social exchange theory concepts to the field of employee turnover intentions is examined (Biron and Boon, 2013). According to the findings of various studies, social mechanisms may help retain employee based on social reciprocity in the health care sector (Biron and Boon, 2013). The social exchange context of work, which refers to an individual’s exchange connections with coworkers and superiors, appears to be missing from the discussion of how retention may (or may not) be related to turnover (Blau, 1964). To fill this gap, this study hypothesis that two elements of the social exchange context, namely Job stress and State anger, influence the employee turnover intentions relationships (Cole et al., 2002). This study have chosen these elements because they draw into the reciprocal and interdependent connections that exist between employees and their supervisors and coworkers (Cole et al., 2002; Uhl-Bien and Maslyn, 2003; Cropanzano and Mitchell, 2005; Koster et al., 2007).

**FIGURE 1:**
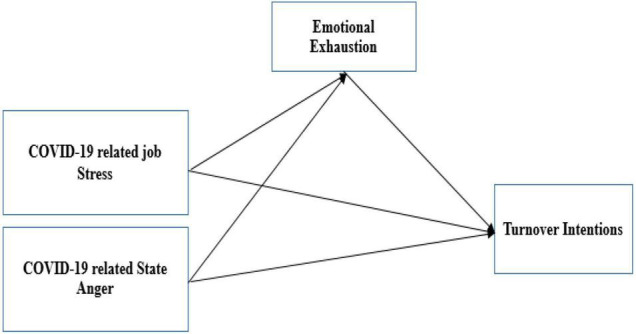
Conceptual framework.

SET supports the abovementioned phenomenon. This theory assumes that an individual’s social behavior occurs as a result of the costs and benefits associated with their current job. In this study, COVID-19-related job stress acts as a cost that nurses bear during this pandemic. When nurses compare these interactional factors, they tend to form their intentions to stay at the workplace or leave the workplace depending upon the weighting of the costs and benefits. When benefits outweigh the costs, nurses want to stay at their workplace; when costs are higher than the benefits (job related stress during COVID-19), then nurses form their intentions to leave their workplace. Due to this pandemic, nurses are suffering from a higher level of mental stress, which leads to turnover intentions. The model of the study is shown in Figure 1.

## Materials and Methods

### Samples and Data Collection

This study used a cross-sectional design, which was conducted to assess nurses’ turnover intentions during the outbreak of COVID-19, and were exposed to the threat of COVID-19-related job stress and state anger.

This study used quantitative approach. The rational behind using quantitative approach is that past quantitative studies have concluded that front line health care workers treating corona infected patients were more likely to go through psychological issues such as stress, restlessness, anxiety and depression (Liu et al., 2020; Xu W. et al., 2021; Xu X. et al., 2021).

Nurses were selected from government-designated hospitals in Islamabad and Rawalpindi, Pakistan, from March 16 to April 30, 2020, by using convenience sampling. The Rational for using convenient sampling technique is that the population for current study was easy for accessibility. Similarly, convenient sample is the most suitable sampling technique for disastrous situation. This sample is more preferred for clinical type of cases, where the participants are placed near hospitals or other medical units (Stratton, 2021). Nurses were selected from the high-risk areas; such as ICUs, isolation wards, emergency departments, respiratory wards, and infection control offices. The informed consent was taken from the chief director of nursing services and from the nurses. The participants were ensured regarding their privacy and confidentiality. A total of 335 nurses were selected for this study. Out of 335 nurses, 318 responses were collected (response rate 92%). Unanswered questionnaires were excluded, and normality was established, and the final analysis was conducted by using the 301 questionnaires.

## Measurement Scales

### COVID-19-Related Job Stress

COVID-19-related job stress was assessed by measuring the pressure from time and anxiety with a scale developed by Parker and DeCotiis (1983). In order to limit job stress within the context of COVID-19, the phrase “caused by SARS-COVID-19” was added to each item. The scale contained nine items and was answered using a five-point Likert scale ranging from 1 (strongly disagree) to 5 (strongly agree). A sample item is “I have felt fidgety or nervous as a result of my job caused by SARS-COVID-19.”

### COVID-19-Related State Anger

This study adopted state anger from the subscale of the State-Trait Anger Expression Inventory (STAXI; Spielberger and Sydeman, 1994). This scale was used to measure how nurses experienced and felt about working as a nurse during the COVID-19 outbreak. Seven items were used to gauge state anger on a five-point Likert scale. A sample item is “I was furious.”

### Emotional Exhaustion

Emotional exhaustion was measured by using the emotional subscale of the Maslach Burnout Inventory-General Survey (MBI; Schaufeli et al., 1996). The scale consisted of five items, which measured being emotionally overextended and drained by one’s work. A sample item is “I felt tired when I got up in the morning and had to face another day on the job.” Responses were assessed using a five-point Likert scale.

### Turnover Intentions

Nurses’ turnover intentions were assessed by using the adapted scale from Farh et al. (1998). The scale consisted of four items and was measured using a five-point Likert scale. A sample item is “I often think of quitting my present job.”

## Data Analysis and Results

In order to test the proposed model, we carried out multiple tests. First, demographic characteristics and descriptive statistics of participants were assessed. Furthermore, confirmatory factor analysis (CFA) was carried out to validate the measurement model by verifying the goodness of fit indices, convergent validity and discriminant validity using SPSS and AMOS 24. Table 1 presents the demographic characteristics; and Table 2 provides the descriptive statistics of the sample. To test the hypotheses, this study used structural equation modeling through Analysis of Moment Structure (in AMOS) to investigate the direct and indirect effects, in line with Obeidat et al. (2016) and Shah and Beh (2016).

**TABLE 1:** Demographic characteristics.

Variables	Frequency	Percentage
**Age**		
20–30	87	28.9
31–40	149	49.5
41–50	56	18.6
51–60	9	3.0
**Qualification**		
Undergraduate	22	7.3
Graduate	128	42.5
Masters	151	50.2
**Experience**		
Fresh	39	13.0
1–5 years	84	27.9
6–10 years	101	33.6
11–20 years	57	18.9
21–30 years	14	4.7
30 and above	6	2.0

**TABLE 2:** Descriptive statistics.

S. No	All variables	Mean	Standard deviation	1	2	3	4
**1**	**State anger**	3.55	(0.80)	(0.881)			
**2**	**Emotional exhaustion**	3.61	(0.80)	0.689**	(0.849)		
**3**	**Turnover intentions**	3.60	(0.82)	0.543**	0.617**	(0.865)	
**4**	**Job stress**	3.66	(0.74)	0.519**	0.644**	0.594**	(0.902)

** Correlation is significant at the 0.01 level (two-tailed). Values in bold are the Cronbach’s alphas.

### Demographic Characteristics and Descriptive Statistics

Table 1 presents the demographic characteristics. It is resulted that majority of respondents were male (62.8%), with the age group of 31–40 comprising the highest proportion of respondents (49.5%). Furthermore, the majority of respondents held a Master’s degree (50.2%) and 6–10 years of work experience.

Further, Table 2 presents the descriptive statistics with means, standard deviation, reliability statistics (Cronbach’s alpha), and intercorrelations. In Table 2, the Cronbach’s alpha values for all variables were greater than the minimum threshold of 0.70, which supports the notion that all the variable instruments used are reliable. Furthermore, it is evident from Table 2 that the relationships among the predictor, mediator, and outcome variables were significantly positively correlated; thus, resulting in preliminary support for the study’s hypotheses.

### Measurement Model

We performed the CFA in order to confirm the factor structure and model fitness of our proposed model. We tested the complete proposed model using CFA with all four variables. We correlated the few error terms of the variables and dropped one item from state anger due to a low factor loading. After taking these measures, the model fitness of indices for the four-factor model showed an adequate model fit by fulfilling the threshold criteria (Hu and Bentler, 1999). Chi-square/df = 2.351, RMSEA = 0.051, GFI = 0.912, CFI = 0.930. Moreover, Tables 3, 4 present the convergent and discriminant validity (Anderson and Gerbing, 1988; Hair et al., 2010). As shown in Table 3, the average value extracted (AVE) for all variables is greater than 0.5, and all factor loading values are higher than 0.50, meeting the threshold for convergent validity (Hair et al., 2010). Moreover, as seen in Table 4, which presents the discriminant validity, the square root of AVEs are greater than the intercorrelations. Hence, discriminant validity is also established for all constructs. In order to check the multicollinearity, the variance inflation factor (VIF) was used and ranged from 1.67 to 4.52 (below 10), which showed no multicollinearity issues.

**TABLE 3:** Construct validity.

Construct	Dimension number	Factor loading	AVE	CR	Cronbach’s alpha
**State anger (SANG)**	SANG 1	0.80	0.59	0.89	0.88
	SANG 2	0.88			
	SANG 3	0.83			
	SANG 4	0.75			
	SANG 5	0.74			
	SANG 6	0.63			

**Emotional exhaustion (EE)**	EE 1	0.84	0.53	0.85	0.84
	EE 2	0.82			
	EE 3	0.72			
	EE 4	0.64			
	EE 5	0.61			

**Turnover intentions (TOI)**	TOI 1	0.77	0.62	0.87	0.86
	TOI 2	0.86			
	TOI 3	0.78			
	TOI 4	0.75			

**Job stress (JS)**	JS 1	0.60	0.51	0.89	0.90
	JS 2	0.60			
	JS 3	0.59			
	JS 4	0.66			
	JS 5	0.79			
	JS 6	0.78			
	JS 7	0.78			
	JS 8	0.79			
	JS 9	0.72			

**TABLE 4:** Discriminatory validity.

	CR	AVE	MSV	MaxR(H)	SANG	JS	EE	TOI
SANG	0.898	0.597	0.462	0.912	0.773			
JS	0.899	0.501	0.475	0.910	0.511	0.707		
EE	0.850	0.535	0.508	0.872	0.680	0.689	0.731	
TOI	0.870	0.626	0.508	0.877	0.491	0.664	0.713	0.791

### Structural Model

We tested the hypotheses through two steps using structural equation modeling (in AMOS). First, H1, H2, H3, H4, and H5 were tested, and the model was subjected to multiple satisfactory goodness of fit indices values (χ2 = 351.484, df = 139, χ2/df = 2.529, RMSEA = 0.071, GFI = 0.927, NFI = 0.905, RFI = 0.911, IFI = 0.940, TLI = 0.926, CFI = 0.939), as suggested by Hair et al. (2010). The impact of COVID-19-related job stress on turnover intentions, COVID-19-related state anger on turnover intentions, COVID-19-related job stress on emotional exhaustion, COVID-19-related state anger on emotional exhaustion, and emotional exhaustion on turnover intentions were statistically significant (see Table 5), hence supporting H1, H2, H3, H4, and H5.

**TABLE 5:** Regression results of the structural model and hypotheses test outcomes.

Hypothesis	Predicted relationship	Standard path loadings	Standard Error	*t*-value	*P*-value	Decision
H1	JS → TOI	0.55	0.078	6.888	0.001	Supported
H2	STAN → TOI	0.20	0.101	3.261	0.002	Supported
H3	JS → EE	0.46	0.095	6.513	0.001	Supported
H4	STAN → EE	0.44	0.089	6.418	0.009	Supported
H5	EE → TOI	0.49	0.093	4.997	0.008	Supported

*JS, job stress; STAN, state anger; TOI, turnover intentions; EE, emotional exhaustion. Goodness-of-fit: χ2/df = 2.529, RMSEA = 0.071, GFI = 0.939, CFI = 0.950.*

Moreover, in the next step, H6 and H7, which proposed indirect effects, were also subjected to multiple satisfactory goodness of fit indices (χ2 = 642.711, df = 235, χ2/df = 2.735, RMSEA = 0.076, GFI = 0.900, NFI = 0.901, RFI = 0.923, IFI = 0.912, TLI = 0.928, CFI = 0.911). The conceptual model proposed that COVID-19-related job stress impacts nurses’ turnover intentions through a mediator (i.e., emotional exhaustion). The standardized estimates along with their corresponding 95% confidence intervals, computed across 5,000 bootstrapped samples (Jose, 2013), are given in Table 6.

**TABLE 6:** Standardized mediation effects: Parameter estimate and bootstrap percentile method confidence intervals.

Hypothesis	Parameter	Estimate	Lower bound	Upper bound	*P* value	Decision
H6	Panel I^a^	0.229	0.138	0.328	0.012	Supported
	JS → EE → TOI					
H7	Panel II^b^	0.220	0.143	0.332	0.009	Supported
	STAN → EE → TOI					

*JS, job stress; STAN, state anger; TOI, turnover intentions; EE, emotional exhaustion. ^a^Goodness-of-fit: χ2/df = 2.529, RMSEA = 0.071, GFI = 0.939, CFI = 0.950. ^b^Goodness-of-fit: χ2/df = 2.735, RMSEA = 0.076, GFI = 0.900, CFI = 0.911.*

## Discussion and Conclusion

The purpose of this research was to examine the effect of COVID-19-related job stress and COVID-19-related state anger on nurses’ turnover intentions directly and indirectly through the mediating effect of emotional exhaustion. In particular, COVID-19-related job stress and COVID-19-related state anger were measured for nurses who experienced the traumatic event of COVID-19. The findings suggest that COVID-19-related job stress has a positive and significant effect on nurses’ turnover intentions. Moreover, COVID-19-related job stress induced nurses’ turnover intentions. Thus, the continuous inflow of patients during the emerging situation caused by the outbreak of such an infectious disease – that is, COVID-19 – caused job stress, which in turn increased nurses’ turnover intentions. These findings are consistent with previous research studies that have reported that nurses’ exposure to traumatic events causes job stress (Hinderer et al., 2014; Li et al., 2014).

The current study established the effect of job-related stress, state anger on turnover intentions among nurses during COVID-19: The mediating role of emotional exhaustion. The results of the study revealed that job related stress and state anger had a direct and indirect influence on turnover intention, with emotional exhaustion playing a mediating effecting on turnover intention (Ferguson et al., 2020). This study has increased the body of knowledge by investigating emotional exhaustion as mediating variable between job related stress, state anger and turnover intention.

In addition, various research studies on SARS show that such a disease causes state anger in nurses (Robertson et al., 2004; Fiksenbaum et al., 2007). Similarly, COVID-19-related state anger was found to have a positive effect on nurses’ turnover intentions in the current study, which is consistent with previous research studies (Greenglass et al., 2003; Fiksenbaum et al., 2007). Given the fact that COVID-19 is novel and has increased the level of uncertainty among nurses, it has triggered a level of state anger and, in turn, has led to increased turnover intentions.

Although there have been a number of research studies investigating the effect of job stress on turnover intentions, an important contribution of this study is that it investigated such relationships during the COVID-19 outbreak – specifically the effect of COVID-19-related job stress on turnover intentions (Hinderer et al., 2014; Li et al., 2014). Moreover, this study is the first to investigate nurses’ turnover intentions during the COVID-19 outbreak and to investigate the effect of COVID-19-related state anger on nurses’ turnover intentions. As such, our study adds to the body of knowledge by contributing to the literature specifically related to the impact of COVID-19-related job stress and state anger on nurses’ turnover intentions.

Similarly, the present results show that emotional exhaustion mediates the relationship between COVID-19-related job stress and turnover intentions. Our results revealed that COVID-19-related job stress increased emotional exhaustion, which in turn led to increased turnover intentions, consistent with previous research studies (e.g., Stordeur et al., 2001; Golparvar et al., 2010; Golparvar, 2016). Moreover, we found that there are underlying mechanisms that indirectly relate to COVID-19-related job stress–turnover intentions. Emotional exhaustion is an underlying mechanism that accounts for this relationship. Emotional exhaustion positively affects turnover intentions (Babakus et al., 2008). Thus, when there is COVID-19-related job stress, nurses are emotionally exhausted, which leads to increased turnover intentions. Building upon these studies, we suggest that emotional exhaustion is not only predicted by COVID-19-related job stress but also affects turnover intentions. Another interesting intervening mechanism that this study examined was the mediating role of emotional exhaustion between COVID-19-related state anger and turnover intentions. This research is pioneering in that it has investigated the mediating role of emotional exhaustion in the COVID-19-related state anger and turnover intention relationship – contributing emotional exhaustion as a new mediating mechanism as emotional exhaustion is one’s feeling of overload and of being drained. The COVID-19-related state anger increases the emotional exhaustion of nurses, which in turn increases their turnover intentions (Oyeleye et al., 2013; Thanacoody et al., 2014; Nazir et al., 2016).

The last contribution of this study is its theoretical contribution; this study extends the COVID-19-related literature by introducing COVID-19-related job stress and COVID-19 related state anger in our model. This study provides empirical evidence from a developing country – Pakistan – from hospitals working under the threat of COVID-19 and targeting nurses who are working with COVID-19-related job stress and COVID-19-related state anger.

## Implications, Limitations and Future Directions

### Implications

The stud results have some significant implications for health care leaders and hospital administrations specially to deal with emergency kind of situations like COVID-19 pandemic. During the times of public health emergencies, health care Units must prioritize the importance to understand the mental health response of front-line fighters. The health care administrators need to revise their selection criteria by focusing on factors such as psychological state, emotional stability, coping with stress of nurses hired during the time of crises or emergencies. Further it is suggested that health care administrators should properly define the sensible work schedules of front-line health workers. More medical equipment’s and facilities should be provided to the health care workers so that they feel more valuable to the organization which will ultimately reduce their intentions to quit. Lastly, during the crises of COVID-19 pandemic hospitals should provide more social and psychological support to their health care workers.

During COVID-19, nursing managers need to pay attention to the turnover intentions of nurses who are facing the issue on the front line, as patients are initially treated by nursing staff. The results of this study are expected to provide relevant information about nurses’ turnover intentions in connection with emerging outbreaks of infectious diseases, such as COVID-19, and contribute to strategies and programs with a view to reducing turnover intentions. To reduce nurses’ turnover intentions, efforts need to be made by nursing managers to reduce job stress, improve the hospital’s resources for treatment, and promote support from hospital management in terms of providing thorough infection protection and education on the COVID-19 infection in regular nursing on-the-job training. Furthermore, the findings of this study provide valuable insights for hospitals and nursing management into nurses’ retention following a COVID-19 outbreak. Moreover, it is important for hospital management to develop effective and systematic management programs that cater to COVID-19-related job stress and state anger in order to reduce turnover intentions. Furthermore, such systematic management programs could be useful tools for observing and avoiding turnover intentions in preparing against potential future infectious disease outbreaks. Moreover, coping management strategies should be introduced by management to reduce turnover intentions related to epidemic or pandemic outbreaks. This research indicates to nursing managers that a hospital’s long-term strategy should incorporate educational programs for management to provide adequate support to nurses.

### Limitations and Future Recommendations

Like all researches, the present study has a range of limitations, each offering directions for future research. First, this study was cross-sectional in nature; future studies should consider a longitudinal design or experimental studies.

Second, this research was carried out in Pakistani hospitals, providing the context of a developing country. Future studies can be conducted in other countries or in different cities to further validate the proposed relationships under the special circumstances of the COVID-19 pandemic. Third, this study adds to the existing literature by considering the impact of COVID-19-related stress and state anger on turnover intentions; further work is required to address a number of possible factors; such as coping strategies, self-efficacy, social support, job satisfaction, that trigger nurses’ turnover intentions. Fourth, this research study provides empirical evidence from Pakistan, a developing country; future studies should focus on developed countries, cross-cultural, cross-country, and compare nurses’ experiences of COVID-19 and their turnover intentions.

## Data Availability Statement

The raw data supporting the conclusions of this article will be made available by the authors, without undue reservation.

## Ethics Statement

The studies involving human participants were reviewed and approved by the Research Ethics Committee of Bahria University. The patients/participants provided their written informed consent to participate in this study.

## Author Contributions

SS and AH: conceptualization. SS and NR: methodology. SS, AH, and NR: software and writing – original draft preparation. SS: formal analysis. NR: investigation. AM and JJ: validation and resources. AM: data curation. AM, NR, and JJ: writing—review and editing. SS and JJ: supervision. AH and JJ: project administration. AM and JJ: funding acquisition. All authors have read and agreed to the published version of the manuscript.

## Conflict of Interest

The authors declare that the research was conducted in the absence of any commercial or financial relationships that could be construed as a potential conflict of interest.

## Publisher’s Note

All claims expressed in this article are solely those of the authors and do not necessarily represent those of their affiliated organizations, or those of the publisher, the editors and the reviewers. Any product that may be evaluated in this article, or claim that may be made by its manufacturer, is not guaranteed or endorsed by the publisher.
